# Adenoid Cystic Carcinoma of the Male Breast: An Exceptionally Rare Presentation

**DOI:** 10.1155/crip/5830252

**Published:** 2026-05-14

**Authors:** Ensiyeh Bahadoran, Afsaneh Yakhforoshha, Farzaneh Abbasi, Fatemeh SamieeRad

**Affiliations:** ^1^ Cellular and Molecular Research Center, Research Institute for Prevention of Non-Communicable Disease, Qazvin University of Medical Sciences, Qazvin, Iran, qums.ac.ir; ^2^ School of Medicine, Qazvin University of Medical Sciences, Qazvin, Iran, qums.ac.ir; ^3^ Department of Pathobiology, School of Medicine, Qazvin University of Medical Sciences, Qazvin, Iran, qums.ac.ir

**Keywords:** adenoid cystic carcinoma, case report, cribriform subtype, male breast cancer, rare breast tumors, solid-basaloid subtype

## Abstract

**Background:**

Adenoid cystic carcinoma (ACC) of the breast is an exceedingly rare malignancy, accounting for less than 0.1% of all breast cancers. It typically affects postmenopausal women and is exceptionally uncommon in men, with fewer than 20 cases reported in the literature. Histologically, ACC is a biphasic tumor composed of epithelial and myoepithelial cells with distinct cribriform, tubular, and solid‐basaloid subtypes. Awareness of this condition in men is essential to prevent misdiagnosis and guide appropriate management.

**Case Presentation:**

We report the case of a 40‐year‐old man who presented with a painful right breast mass that had been present for 3 years. Ultrasonography revealed multiple solid lesions, and histopathological examination following surgical excision confirmed ACC with cribriform and solid patterns. Immunohistochemistry results were negative for ER, PR, HER2, and p53, with a Ki‐67 index of 17%. Although a chest CT scan revealed suspicious bilateral pulmonary nodules, FDG‐PET/CT did not reveal evidence of distant metastasis. Subsequent total mastectomy after 3 months revealed cribriform ACC without lymph node involvement. Due to imaging findings suggestive of possible local recurrence or residual disease, the patient was treated with adjuvant radiotherapy.

**Conclusion:**

This case highlights the importance of considering ACC in the differential diagnosis of breast masses in male patients. Given the rarity of this tumor in males, accurate histopathological and immunohistochemical diagnoses are critical to avoid misclassification. Although the overall prognosis is favorable, close follow‐up is warranted because of the potential for recurrence and metastases.

## 1. Introduction

Adenoid cystic carcinoma (ACC) is comparatively uncommon in the breast, prostate, esophagus, trachea, and other locations; however, it typically affects the salivary glands (1). The incidence of ACC, a rare form of breast cancer, is less than 0.1% (2). It primarily affects postmenopausal women, and there have only been 18 cases documented in men; the condition is likely to be missed or misdiagnosed (3, 4). For example, one study noted that up to 50% of breast ACC cases were initially misclassified (2). According to recent classifications, ACC of the breast is currently regarded as a distinct special‐type carcinoma and is classified within the group of salivary gland–type tumors of the breast (5).

A hallmark histologic feature of ACC is the presence of two distinct types of luminal structures formed by a dual population of luminal epithelial and abluminal myoepithelial–basal cells (6). True glandular lumina are lined by luminal ductal epithelial cells with round nuclei and eosinophilic cytoplasm and often contain mucinous material. These cells typically express low‐molecular‐weight cytokeratins, including CK7 and CK8/18, as well as epithelial markers such as EMA and CD117 (c‐Kit). In contrast, pseudolumina result from stromal invaginations and contain dense eosinophilic basement membrane‐like material. They are surrounded by myoepithelial or basal cells, which show scant cytoplasm and are immunoreactive for basal cytokeratins (e.g., CK5/6, CK14, and CK17) and myoepithelial markers such as p63, smooth muscle actin, calponin, and S‐100 protein (7). At the molecular level, MYB, an oncogenic transcription factor and progenitor cell regulator, is frequently overexpressed in ACC (8). The most common cause of MYB overexpression is a t (6;9) (q22–23; p23–24) translocation that leads to MYB::NFIB fusion. Other causes include MYB amplification or changes in the MYB paralog MYBL1 (9). MYB rearrangements are observed in approximately 22.6% of patients with breast ACC (4).

There are three histological subtypes of breast ACC: cribriform, solid, and tubular. The most prevalent subtype is cribriform, characterized by glandular epithelial and myoepithelial/basal cells forming cancer nests with true and pseudocystic spaces (4). Histologically, solid‐basaloid ACC is characterized by a basaloid cytological appearance, comprising rounded nests and trabeculae embedded in a fibromyxoid stroma (6). In addition to conventional ACC subtypes, ACC with high‐grade transformation has been described. It is a rare variant and formerly referred to as “dedifferentiated ACC.” It is characterized by the abrupt emergence of a high‐grade carcinoma component with loss of the typical biphasic ductal–myoepithelial architecture, increased mitotic activity, necrosis, and marked nuclear atypia. It is associated with a significantly more aggressive clinical course, including higher rates of lymph node and distant metastases and poorer overall survival compared with conventional ACC (10, 11). ACC of the breast is a triple‐negative breast cancer (TNBC) that lacks ER, PR, and HER2 expression and resembles the basal‐like TNBC subtype. In contrast to the poor outcomes of most TNBC phenotypes, this cancer has a favorable prognosis because regional lymph node involvement and distant metastases occur infrequently (12). It has a high recurrence rate because of frequent involvement of the resection margin. Moreover, even if local recurrence occurs, more than half of the patients can be cured with subsequent mastectomy (13). The 5‐year, 10‐year, and 15‐year relative survival rates of this disease are 98.1%, 94.9%, and 91.4%, respectively (2). Patients with solid‐basaloid or high‐grade subtypes are more likely to experience distant metastases and local recurrence (3) and may require closer follow‐up and consideration of additional therapy. The primary treatment for breast ACC is surgery, followed by radiotherapy (3).

Herein, we present the case of a 40‐year‐old man with ACC of the breast, showing both cribriform and solid subtypes. Given its rarity in males, awareness of this entity among oncologists and pathologists is essential to avoid misdiagnosis and ensure appropriate management of the condition.

## 2. Case Presentation

A 40‐year‐old man with no history of breast cancer presented with a palpable mass in the right breast. He stated that he first noticed the mass approximately 3 years ago, at which time it was painless; however, in recent years, it had become painful upon palpation. However, the patient did not report any systemic symptoms or fatigue. Four years prior, the patient had undergone surgery for gynecomastia, during which a mass measuring 6.5 × 5.5 × 2 cm was excised. Macroscopic examination revealed that the specimen consisted of several pieces of lobulated yellow and creamy‐colored tissue with an elastic consistency. Microscopic analysis revealed a benign neoplastic lesion originating from the apocrine glands, characterized by proliferative glandular structures predominantly lined by a single layer of cuboidal, mostly acidophilic cells.

Physical examination revealed a firm, tender mass in the retroareolar region of the right breast with no signs of retraction or discharge. Mild axillary lymphadenopathy was observed bilaterally.

Breast ultrasonography revealed the following findings: In the right breast, a solid hyperechoic lesion with well‐defined margins measuring 11 × 9 mm was identified in the retroareolar region, likely corresponding to the clinically palpable mass (Figure 1a). Two additional solid hypoechoic lesions with spiculated margins were observed at the 3 o’clock position, measuring 7 × 6 mm and 17 × 11 mm, and located 1 and 2 cm from the nipple, respectively (Figure 1b). A solid isoechoic lesion with well‐defined margins measuring 10 × 6 mm was observed at the 4 o’clock position, 2 cm from the nipple (Figure 1c). A vertically oriented isoechoic solid lesion with partial angular margins measuring 11 × 12 mm was identified in the 6 o’clock subareolar region (Figure 1d). No space‐occupying lesions, cysts, or stellate masses were observed in the left breast. The skin and subcutaneous tissues showed normal thickness, without any evidence of abnormal thickening. Two reactive lymph nodes with a maximum diameter of 12 × 5 mm were detected in both axillary regions. Based on these findings, the lesion was assigned a BIRADS Category 4, and tissue sampling via core needle biopsy or excision surgery was recommended.

Figure 1Right breast ultrasonography. (a) A hyperechoic lesion with well‐defined margins in the retroareolar region (11 × 9 mm). (b) A hypoechoic spiculated lesion with spiculated margins at the 3 o’clock position (17 × 11 mm). (c) A solid isoechoic lesion with well‐defined margins at the 4 o’clock position (10 × 6 mm). (d) A solid lesion isoechoic with angular margins in the subareolar 6 o’clock region (11 × 12 mm).

**Figure:**
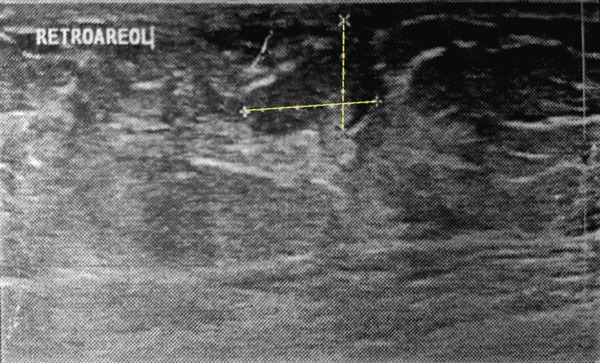
(a)

**Figure:**
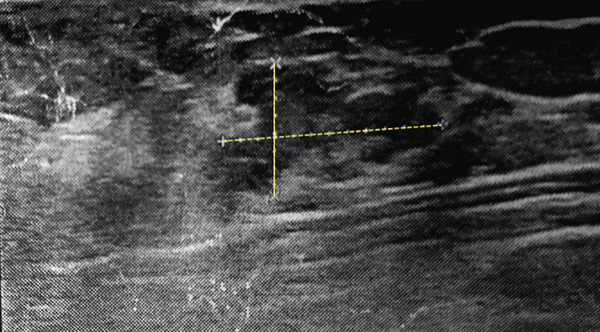
(b)

**Figure:**
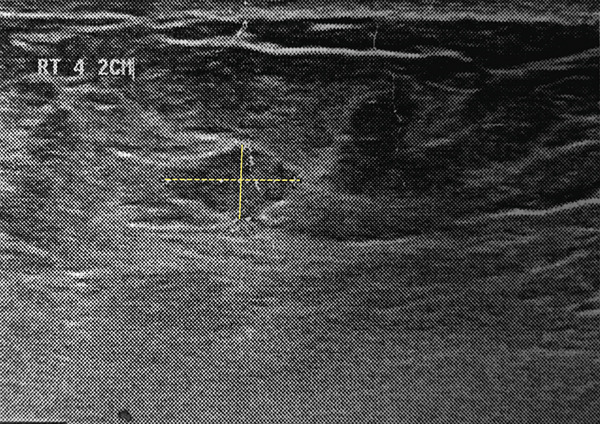
(c)

**Figure:**
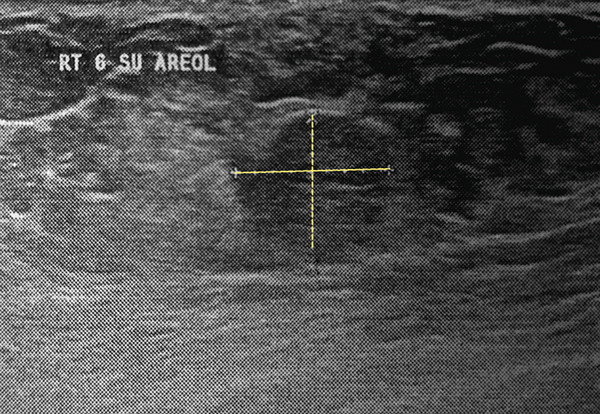
(d)

**Figure 2:**
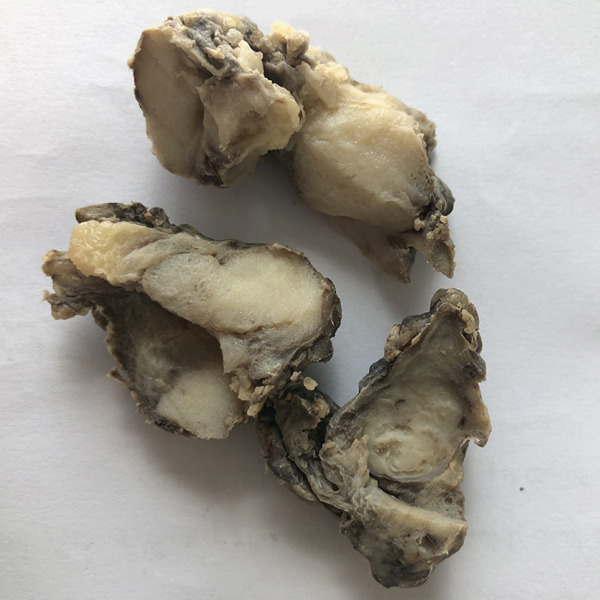
Gross pathology of the excised breast tissue. Multiple pieces of irregular brown tissue, with a total mass of 8 × 6 × 3 cm. Serial sectioning showed multiple creamy, lobulated masses with a homogenous soft to rubbery consistency.

Subsequently, the patient underwent surgical excision of the right breast lesion. Gross examination of the surgical specimen revealed multiple pieces of irregular brown tissue with a total mass of 8 × 6 × 3 cm (Figure 2). Serial sectioning showed multiple creamy, lobulated masses with a homogeneous soft to rubbery consistency.

Microscopic examination demonstrated a malignant infiltrative neoplasm with a lobular architecture, characterized by cribriform nests and small solid epithelial islands, along with numerous cylindrical structures containing homogenous eosinophilic material. The tumor cells were cuboidal, with high nuclear‐to‐cytoplasmic ratios and hyperchromatic nuclei. No perineural invasion was observed (Figures 3a, 3b, and 3c). Immunohistochemical analysis revealed that the tumor was negative for ER, PR, HER2, and P53, with a Ki‐67 proliferation index of 17%. Based on the histopathological and IHC findings, the final diagnosis was ACC of the breast. Molecular genetic testing for MYB or MYBL1 rearrangements was not performed in this case due to the limited availability of molecular diagnostic facilities.

Figure 3Histopathological examination of the adenoid cystic carcinoma (ACC) of the breast. (a, b) A lobulated infiltrative neoplasm with cribriform nests and small solid epithelial islands (H&E, 40×, 100×). (c) Cylindrical structures filled with eosinophilic basement membrane‐like material and cuboidal tumor cells. Individual tumoral cells have high nuclear‐to‐cytoplasmic ratios and hyperchromatic nuclei (H&E, 400×).

**Figure:**
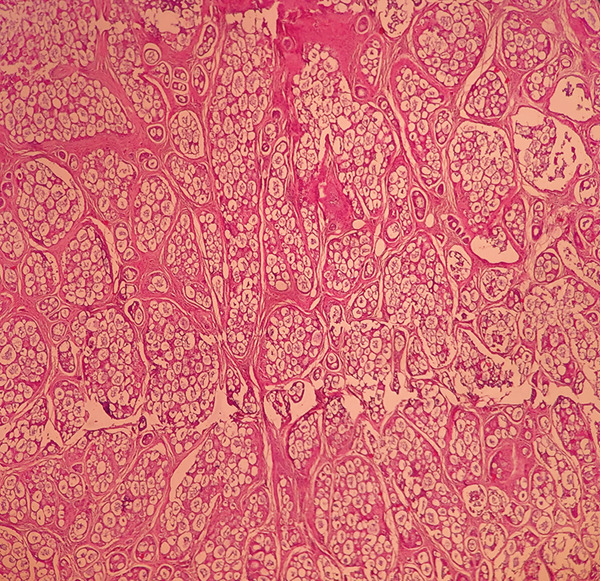
(a)

**Figure:**
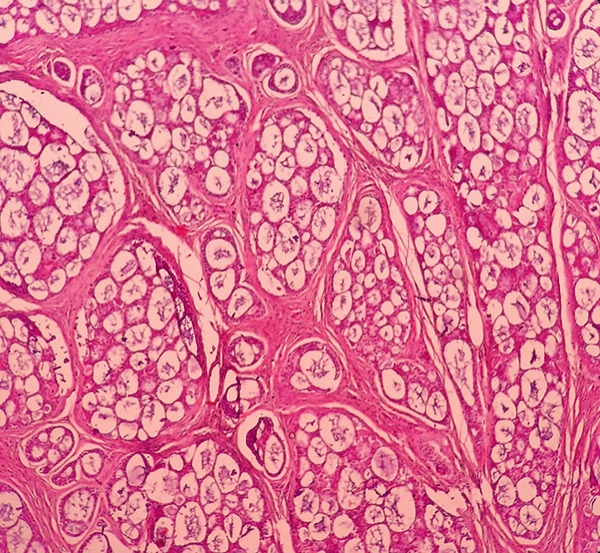
(b)

**Figure:**
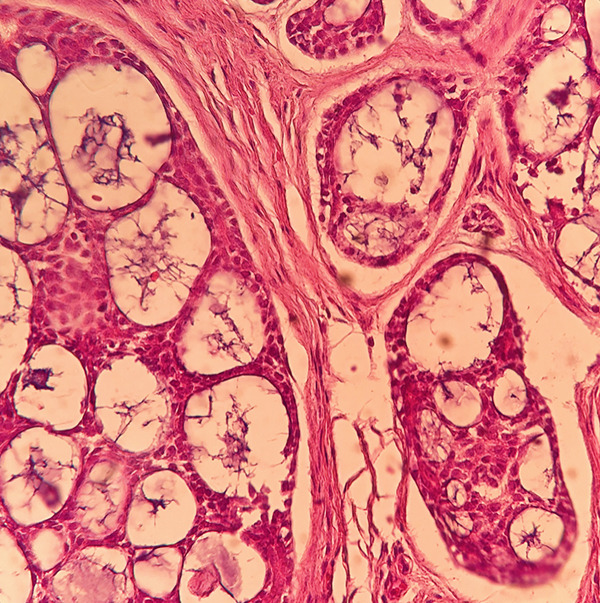
(c)

In a follow‐up evaluation 2 months later, breast MRI with and without contrast of the right breast revealed a 44 × 14 mm irregular nonmass enhancement with associated skin involvement, skin retraction, and enhancement of the pectoralis major fascia, which were considered to be either postoperative changes or remnants of the primary tumor.

A spiral chest CT scan with intravenous contrast demonstrated a few scattered bilateral pulmonary nodules, the largest measuring 7 mm in the lingula, along with bilateral upper and lower lobe ground‐glass reticulonodular infiltrations. Chest wall assessment revealed postoperative skin depression of the right breast, loss of subcutaneous fat replaced by soft tissue density, and an 8 mm nodular soft tissue lesion, which warranted follow‐up due to the need to rule out metastasis.

Consequently, an FDG‐PET/CT scan was performed, which revealed a soft tissue lesion with mild FDG uptake in the right anterior chest wall (SUVmax: 3.47, measuring approximately 31 × 15 mm). No other abnormal FDG uptake was observed in the chest wall or the left breast.

The patient subsequently underwent a modified radical mastectomy. Pathological examination of the resected tissue revealed a 1.5 cm cribriform tumor, and all three excised lymph nodes were free of tumor involvement.

Although the FDG‐PET/CT scan did not reveal evidence of metastatic disease, given the limited sensitivity of this imaging modality in detecting metastases smaller than 1 cm, and based on the recommendation of the oncologist, the patient was treated with radiotherapy. At 10 months of follow‐up after completion of radiotherapy, the patient was asymptomatic, and no clinical or radiological evidence of disease recurrence was identified.

## 3. Discussion

ACC has been reported in the salivary glands, external auditory canal, and other parts of the head and neck, uterus, digestive tract, cervix, prostate gland, and skin (12). ACC of the male breast is extremely uncommon. Considering the current and previous cases, there have been 19 reported cases of ACC in the male breast (4).

The incidence of new breast cancer typically occurs 5 years later in men (67 years) than in women (62 years) (14). However, ACC of the breast is more frequently observed in younger male patients. In females, ACC of the breast typically occurs between the ages of 39 and 73 years, with a median age of 60.5 years (15). Including the present case, in males, the age ranged from 13 to 82 years, with a median age of 40 years. The diameter of the lesion varied from 0.6 to 5 cm (16–18). The case reported here is that of a 40‐year‐old male, and the diameter of the lesions ranges from 0.6 to 1.7 cm.

Black males have significantly higher incidence rates than White men for all breast cancer subtypes based on HR/HER2 status (19). However, Asia accounts for over 70% of all documented ACC cases in males over the past 30 years (20). There is a mean of 1 year between the onset of symptoms and diagnosis in male patients with breast cancer. Three main factors that contribute to late presentation were reported to be lack of awareness, embarrassment, and the need for social support (21). Similarly, our case reported the presence of a tumor 3 years ago.

The clinical signs and symptoms of male breast cancer are comparable to those of female breast cancer. Palpable breast lumps, nipple discharge, nipple retraction, and skin changes across the breast, such as redness or dimpling, are typical symptoms. Because men have comparatively less breast tissue, these tumors are frequently easier to detect (22). In contrast, ACC of the male breast most commonly manifests as a firm, palpable subareolar tumor that may be sensitive or painful and is often multifocal. Other symptoms include nipple retraction and skin ulceration. None of the currently available instances have reported breast discharge (20, 23).

The imaging characteristics of ACC in the male breast have not been thoroughly characterized because this condition is rare. Female breast ACC frequently appears on mammography as an irregular, lobulated, and heterogeneous mass with borders that are either unclear or microlobulated (24). On ultrasound, the lesions are characterized by irregular, heterogeneous, or hypoechoic lesions and demonstrate minimal vascularity on color Doppler assessment (25). Compared to the ACC of the female breast, male patients exhibit comparable nonspecific sonographic features, often presenting as masses with clear and unclear boundaries and displaying a mixed or hypoechoic lesion (20, 23, 26). Moreover, they can be solid, nodular, or hypervascular (27). The prominent features of mammography in males are spiculate hyperdense lesions in the subareolar area (23).

The main histological differential diagnoses of ACC of the breast, particularly when it exhibits cribriform or tubular growth patterns, include invasive cribriform carcinoma, invasive tubular carcinoma, and cribriform ductal carcinoma in situ. The histological features of ACC of the breast closely resemble those of its salivary gland counterpart, displaying a range of architectural patterns, including tubular‐trabecular, cribriform, solid‐basaloid, and occasionally microcystic arrangements. These tumors are composed of a characteristic dual‐cell population, inner glandular epithelial cells, typically cuboidal with eosinophilic cytoplasm, round nuclei, and occasional nucleoli, and outer myoepithelial or basal cells, which are spindle‐shaped with scant cytoplasm, hyperchromatic nuclei, and occasional mitotic figures. These two cell layers are consistently observed across various tumor patterns (4, 7). In contrast to ACC of the salivary gland, nerve infiltration is uncommon in ACC of the breast (28). ER, PR, and HER‐2 are not usually expressed in breast ACC. The immunophenotype of ACC of the breast is distinct from those of other TNBCs. Glandular epithelial cells frequently express CK5/6, CK8/18, and CD117, whereas myoepithelial and basaloid cells generally express p63, S‐100, CK5, CK6, CK14, and CK17. In ACCB, Ki‐67 expression is comparatively low (3). ACC of the breast can metastasize to the lungs, bones, liver, brain, and kidneys (12). Distant metastases to the bone and lung have been reported as an initial symptom of ACC of the male breast (27, 29). Surgery with adjuvant radiotherapy improves survival in patients with ACC of the breast (30). Although bilateral pulmonary nodules were observed in this case, the FDG‐PET/CT scan did not show any uptake in these nodules. However, it should be noted that FDG lacks sufficient sensitivity and specificity for nodules less than 1 cm (31); thus, pathological examination and biopsy are required. In this case, given the suspicion of metastasis, the patient’s young age, and clinical judgment, radiotherapy was initiated.

## 4. Conclusion

In conclusion, ACC of the male breast is an extremely rare and often underrecognized malignancy that poses diagnostic and therapeutic challenges. This case highlights the importance of considering ACC in the differential diagnosis of breast masses in men, particularly when they present as subareolar, multifocal, and painful lesions. Histopathological evaluation of the lesion, along with an immunohistochemical profile, is essential for an accurate diagnosis. Given the potential for local recurrence and distant metastasis, a multidisciplinary approach involving surgery and adjuvant radiotherapy, as demonstrated in this case, may improve patient outcomes in the future. Greater awareness among clinicians and pathologists is crucial to avoid delays in diagnosis and ensure timely and appropriate management of this rare entity.

## Author Contributions

E.B.: investigation, visualization, writing—original draft, and writing—review and editing. A.Y.: writing—review and editing. F.A.: writing—original draft and investigation. F.S.: conceptualization, investigation, supervision, visualization, and writing—review and editing.

## Funding

No funding was received for this manuscript.

## Consent

Written informed consent was obtained from the patient to publish this report in accordance with the journal’s patient consent policy.

## Conflicts of Interest

The authors declare no conflicts of interest.

## Data Availability

The authors have nothing to report.
